# Prognostic impact of functional mitral regurgitation prior to left ventricular assist device implantation

**DOI:** 10.1186/s13019-021-01748-9

**Published:** 2022-02-25

**Authors:** Jonas Pausch, Oliver Bhadra, Julian Mersmann, Lenard Conradi, Bjoern Sill, Markus J. Barten, Hermann Reichenspurner, Alexander M. Bernhardt

**Affiliations:** Department of Cardiovascular Surgery, University Heart & Vascular Center Hamburg, Martinistraße 52, 20251 Hamburg, Germany

**Keywords:** Heart failure, Left ventricular assist device, Right heart failure, Functional mitral regurgitation, Mitral valve repair, Mitral leaflet tethering

## Abstract

**Background:**

Functional mitral regurgitation (FMR) is a common finding of advanced heart failure with detrimental effects. The prognostic impact of uncorrected FMR prior to left ventricular assist device (LVAD) implantation remains controversial.

**Methods:**

Between 2016 and 2019 77 patients underwent continuous-flow LVAD implantation at our institution. 34 patients showed FMR ≥ 2 (***MR-group***), whereas 43 patients showed FMR < 2 (***Control-group***). Data was retrospectively analyzed. Primary composite endpoint comprised freedom from death, stroke, pump-thrombosis, major bleeding and right heart failure (RHF) after 1 year.

**Results:**

Baseline characteristics, including the severity of left and right ventricular dysfunction, and periprocedural results were comparable. The overall survival during a mean follow up of 24.9 months was 55.9% in the ***MR-group*** versus 58.1% in the ***Control-group*** (*p* = 0.963), whereas 1-year event-free survival was 35.3% in the ***MR-group*** compared to 44.2% in the ***Control-group*** (*p* = 0.404). RHF within the first postoperative year occurred more frequently in the ***MR-group*** (35.3% vs. 11.6%; *p* = 0.017). Furthermore, RV function was significantly reduced in comparison to baseline values in the ***MR-group***. 12 months after surgery, 74% of patients in the ***MR-group*** were classified as NYHA III in comparison to 24% of patients in the ***Control-group*** (*p* < 0.001).

**Conclusions:**

Preoperative uncorrected FMR prior to LVAD implantation did not affect overall survival, nevertheless it was associated with an impaired RV function and increased incidence of right heart failure during follow-up. Furthermore, preoperative FMR ≥ 2 was associated with persistent symptoms of heart failure.

## Background

**Fig. 1:**
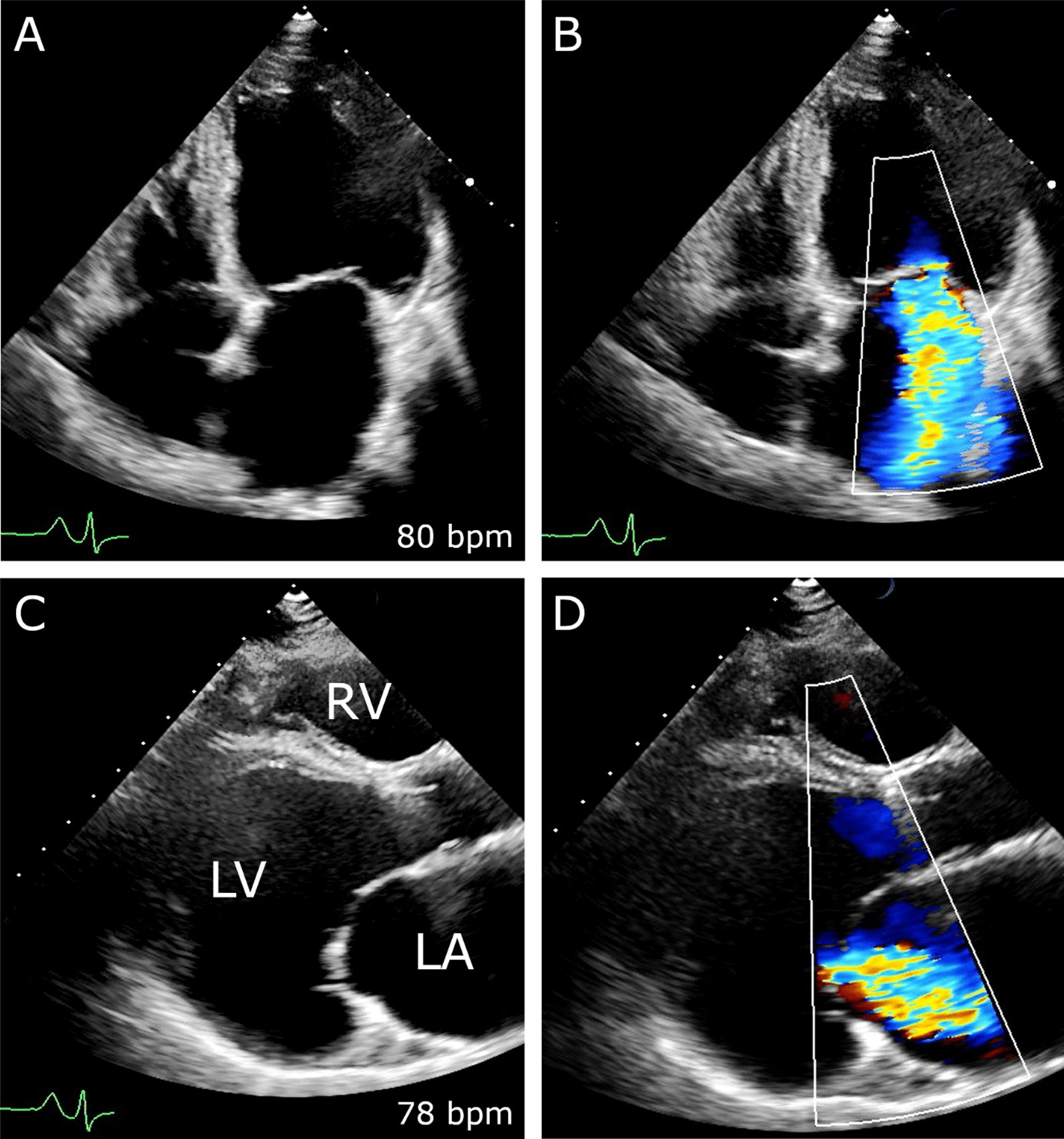
Severe FMR due to left ventricular remodeling. Preoperative transthoracic echocardiogram. Severe functional mitral regurgitation due to advanced LV distortion and mitral leaflet tethering in apical four chamber (**A**, **B**) and parasternal long-axis view (**C**, **D**)

Functional mitral regurgitation (FMR) is a common manifestation of left ventricular (LV) distortion. During the progression of advanced heart failure global and regional LV remodeling results in mitral annulus dilatation along with severe leaflet tethering [1] (Fig. 1). FMR related chronic LV volume overload is associated with an increased diastolic wall stress, an aggravation of LV dilatation and promotes heart failure itself. Furthermore, it is accompanied by an increased rate of adverse cardiac events and mortality, independent of the degree of the underlying LV dysfunction [2].

In addition to optimized guideline-directed medical therapy surgical and percutaneous transcatheter mitral valve repair might be an option for highly-selected heart failure patients with appropriate anatomical features [3–6]. Nevertheless, particularly end-stage heart failure patients with severe FMR due to advanced LV remodeling might not benefit from mitral valve intervention [7].

Continuous flow left ventricular assist device (LVAD) implantation has evolved as an alternative to heart transplantation (HTx) in the treatment of symptomatic advanced heart failure [8–11]. Via LV mechanical unloading long-term LVAD therapy decreases LV dilatation, thereby promoting LV reverse remodeling [12–14] and potentially improving mitral leaflet coaptation [15]. Consequently, preoperative FMR due to advanced LV distortion might be partially resolved via mechanical unloading after LVAD implantation [16, 17]. Furthermore, as uncorrected FMR prior to LVAD implantation does not to affect primary outcome and overall survival after LVAD implantation [18], concomitant routine mitral valve surgery at the time of LVAD implantation is not recommended and currently limited to selected patients [19, 20].

Nevertheless, the prognostic impact of uncorrected FMR prior to LVAD implantation remains controversial [21].

## Patients and methods

### Patients

Pre-, peri- and postoperative data of 77 consecutive patients who underwent continuous-flow LVAD implantation at our department between 2015 and 2018 was retrospectively collected, using a standardized protocol. Patients were selected for LVAD therapy according to current guidelines due to persistent advanced heart failure as bridge to recovery, bridge to heart HTx or destination therapy [22]. Data were retrospectively analyzed, and all patients were categorized according to preoperative FMR severity. Patients with degenerative MR or concomitant mitral valve procedures were not included. Follow-up at our Heart Failure Clinic was complete. The study was approved by our institutional ethics board and is compliant with the ISHLT ethics statement.

### Surgical setup and technique of LVAD implantation

Surgery was performed under general anesthesia in a standard operating room by a dedicated heart failure surgery team, either via full-sternotomy, or minimally invasively using a left-sided thoracotomy approach. Normothermic cardiopulmonary bypass (CBP) was used in all patients. Regardless of the extend of preoperative FMR, no patient underwent any concomitant mitral valve procedure. Concomitant procedures (e.g., aortic valve replacement, tricuspid valve repair) were performed following our institutional standards without major modification during the study period. Implantation of a temporary right ventricular assist device (tRVAD) was done due to unsuccessful weaning from CBP or increased pharmacological inotropic support. Postoperatively phenprocoumon with a target international normalized ratio of 2.0 to 2.5 was administered in addition to acetylsalicylic acid (100 mg per day). All patients underwent a systematic follow-up every 3 to 6 months including a standardized clinical examination, a 6-min walk test (6 MWT), a routine blood test and an echocardiographic assessment at our institution.

### Echocardiography

A standardized echocardiographic assessment was performed preoperatively and during follow-up to evaluate left and right ventricular (RV) function and dimensions [23, 24]. The degree of MR was determined either quantitatively or semi-quantitatively [25]. For the purpose of this study, we defined two study-groups, consisting of patients with FMR ≥ 2 (***MR-group***) and FMR < 2 (***Control-group***) at baseline.

### Endpoints

Primary composite endpoint included freedom from death, stroke, pump-thrombosis, major bleeding and right heart failure (RHF) 1 year after LVAD implantation. Stroke was defined as neurologic deficit and an associated positive cranial computed tomography scan. Pump thrombosis was defined as the combination of an increased energy consumption, an increased calculated forward flow, as well as elevated serum markers of hemolysis. The definition of RHF was adapted from the INTERMACS definition and comprised elevated central venous pressure (CVP) > 16 mmHg or the dilatation of the inferior vena cava leading to clinical manifestations of congestion (e.g., ascites, edema) or increased serum levels of bilirubin [26]. A major bleeding complication was considered whenever urgent transfusion of red blood cells was indicated.

### Statistical analysis

Baseline, perioperative and follow-up variables were collected in a dedicated institutional LVAD database. All patients underwent a systematic follow-up every 4 to 6 months including a standardized clinical examination, a 6 MWT, a routine blood and echocardiographic assessment at our institution. Data are presented as absolute numbers and percentages for categorical variables and mean values ± standard deviation for continuous variables, unless stated otherwise. SPSS 24 software was used for all statistical analyzes. Univariate analysis was performed using t-test for numeric variables (i.e., after confirmation of normal distribution) and chi-square-test/Fisher’s exact test for categorical variables. Kaplan–Meier method was used for the composite endpoint, as well as rates of survival and RHF. Univariate comparisons were performed by log-rank test. Results were considered statistically significant if the *p* value was < 0.05.

## Results

### Study population and patient characteristics

77 consecutive patients suffering from advanced heart failure underwent continuous-flow LVAD implantation at our institution between 2015 and 2018. According to preoperative echocardiography 34 patients (44.2%) showed moderate to severe FMR (***MR-group***) (e.g., Fig. 1), whereas 43 patients (55.8%) showed less than moderate FMR (***Control-group***). Predominantly male patients (84.4%) with a mean age of 54 years were treated (Table 1). Patients in the ***Control-group*** suffered significantly more often from an ischemic cardiomyopathy compared to the ***MR-group*** (i.e., 28 (65.1%) vs. 11 (32.4%); *p* = 0.004). Furthermore, the prevalence of arterial hypertension, previous sternotomy and previous coronary artery bypass grafting (CABG) was significantly higher in the ***Control-group*** (Table 1). Other comorbidities, including atrial fibrillation, previous stroke, chronic obstructive pulmonary disease (COPD), chronic renal failure and previous hemodialysis were comparable in both study-groups (Table 1). All study patients presented with severe symptoms (i.e., NYHA III/IV) of congestive heart failure and elevated serum levels of natriuretic peptide (i.e., 10,336 ± 8360 pg/ml in the ***MR-group*** vs. 9692 ± 8816 pg/ml in the ***Control-group***; *p* = 0.766) (Table 1). Preoperative serum levels of creatinine, glutamic oxaloacetic transaminase (GOT), glutamic pyruvic transaminase (GPT) and bilirubin as signs of secondary end-organ damage due to advanced heart failure, were comparably elevated in both study-groups (Table 1). Short-term mechanical circulatory support until LVAD implantation was necessary in both study-groups (i.e., 9 (26.5%) ***MR-group*** vs. 13 (30.2%); *p* = 0.717) (Table 1).

**Table 1:** Preoperative patient characteristics

Variables	MR-group (n = 34)	Control-group (n = 43)	*p* value
Age (years), mean ± SD	52.0 ± 13.2	56.3 ± 10.9	0.126
Male, n (%)	27 (79.4)	38 (88.4)	0.282
BMI, kg/m^2^, mean ± SD	24.9 ± 4.5	26.9 ± 6.3	0.115
Ischemic cardiomyopathy, n (%)	11 (32.4)	28 (65.1)	0.004
Non-ischemic dilated cardiomyopathy, n (%)	23 (67.6)	15 (34.9)	0.004
Arterial hypertension, n (%)	10 (29.4)	23 (53.5)	0.034
Diabetes mellitus, n (%)	11 (32.4)	14 (32.6)	0.985
COPD > GOLD II, n (%)	4 (11.8)	10 (23.3)	0.194
Atrial fibrillation, n (%)	16 (47.1)	23 (58.1)	0.102
Previous stroke, n (%)	4 (11.8)	5 (11.6)	0.985
Chronic renal failure, n (%)	26 (76.5)	26 (60.5)	0.136
Previous hemodialysis, n (%)	5 (14.7)	6 (14.0)	0.925
Serum creatinin level (mg/dl), mean ± SD	1.6 ± 0.6	1.7 ± 0.7	0.279
Serum NT-proBNP level (pg/dl), mean ± SD	10,336 ± 8360	9692 ± 8816	0.766
Destination therapy, n (%)	12 (35.3)	23 (53.5)	0.113
Serum GOT level (U/l), mean ± SD	73.8 ± 114.7	103.5 ± 237.1	0.482
Serum GPT level (U/l), mean ± SD	80.1 ± 142.6	93.9 ± 159.2	0.691
Serum bilirubin level (mg/dl), mean ± SD	1.3 ± 0.8	1.2 ± 0.8	0.547
Previous short-term MCS, n (%)	9 (26.5)	13 (30.2)	0.717
Previous sternotomy, n (%)	5 (14.7)	16 (37.2)	0.028
Previous CABG, n (%)	3 (8.8)	11 (25.6)	0.026

*BMI* body mass index, *COPD* chronic obstructive pulmonary disease, *GOLD* Global Initiative for Chronic Obstructive Lung Disease, *NYHA* New York Heart Association, *NT-pro-BNP* N-terminal pro-B natriuretic peptide, *GOT* glutamic oxaloacetic transaminase, *GPT* glutamic pyruvic transaminase

Baseline systolic LV and RV functions were similar in both study-groups (i.e., LVEF 19.7 ± 6% ***MR-group*** vs. 21.0 ± 6% ***Control-group***; *p* = 0.341; TAPSE 14.9 ± 4 mm ***MR-group*** vs. 14.9 ± 5 mm ***Control-group***; *p* = 0.979) (Table 2). Severe dilatation of the left ventricle and left atrium could be shown in both study-groups (Table 2). Concomitant aortic regurgitation (AR) was comparable in both study-groups, whereas relevant tricuspid regurgitation (TR) was more frequent in the ***MR-group*** (i.e., 21 (61.8%) vs. 11 (25.6%); *p* = 0.001) (Table 2).

**Table 2:** Preoperative echocardiographic characteristics

Variables	MR-group (n = 34)	Control-group (n = 43)	*p* value
LVEF (%), mean ± SD	19.7 ± 5.7	21 ± 6.4	0.341
LVEDD (mm), mean ± SD	76.0 ± 13.8	70.4 ± 8.7	0.101
TAPSE (mm), mean ± SD	14.9 ± 4.3	14.9 ± 5.2	0.979
LA-volume (ml), mean ± SD	105.7 ± 32.3	95.2 ± 38.0	0.397
Tricuspid regurgitation ≥ 3, n (%)	21 (61.8)	11 (25.6)	0.001
Aortic regurgitation ≥ 2, n (%)	5 (14.7)	4 (9.3)	0.464

*LVEF* left ventricular ejection fraction, *LVEDD* left ventricular end-diastolic diameter, *LA-volume* left atrial volume, *TAPSE* tricuspid annular plane systolic excursion

### Procedural outcome of LVAD implantation

Predominantly full-sternotomy was used (i.e., 79.2%) as surgical access and patients were mainly treated with the Medtronic HVAD device (95%) (Table 3). Normothermic cardiopulmonary bypass (CBP) was used in all patients. Notably, regardless of the extend of preoperative FMR no patient underwent any concomitant mitral valve procedure. The rate of concomitant aortic valve replacement was comparable in both study-groups (5 (14.7%) ***MR-group*** vs. 6 (14.0%) ***Control-group***; *p* = 0.925), however, tricuspid valve repair was performed more frequently in the ***MR-group*** (4 (11.8%) vs. ***Control-group*** 1 (2.3%); *p* = 0.095) (Table 3). Due to unsuccessful weaning from CBP or increased pharmacological inotropic support in addition to echocardiographic signs of RHF, the implantation of a temporary right ventricular assist device (tRVAD) was performed equally in both study-groups (12 (35.3%) in ***MR-group*** vs. 9 (20.9%) in ***Control-group***; *p* = 0.160). The overall duration of the surgery (300 ± 100 min ***MR-group*** vs. 333 ± 94 min ***Control-group***; *p* = 0.155), as well as cardiopulmonary bypass time (155 ± 69 min ***MR-group*** vs. 154 ± 62 min ***Control-group***; *p* = 0.930) were comparable in both study-groups (Table 3). There was no intraprocedural mortality within both study-groups.

**Table 3:** Periprocedural outcome

Variables	MR-group (n = 34)	Control-group (n = 43)	*p* value
Full-sternotomy, n (%)	26 (76.5)	35 (81.4)	0.597
Medtronic HVAD device, n (%)	33 (97.1)	40 (93.0)	0.428
Implantation of tRVAD, n (%)	12 (35.3)	9 (20.9)	0.160
Additional AVR, n (%)	5 (14.7)	6 (14.0)	0.925
Additional TV repair, n (%)	4 (11.8)	1 (2.3)	0.095
Duration of surgery (min), mean ± SD	300.5 ± 100.2	332.9 ± 93.7	0.155
Cardiopulmonary bypass time (min), mean ± SD	155.6 ± 68.7	154.2 ± 61.5	0.930

*tRVAD* temporary right ventricular assist device, *AVR* aortic valve replacement, *TV repair* tricuspid valve repair

### Primary composite study endpoint

**Fig. 2:**
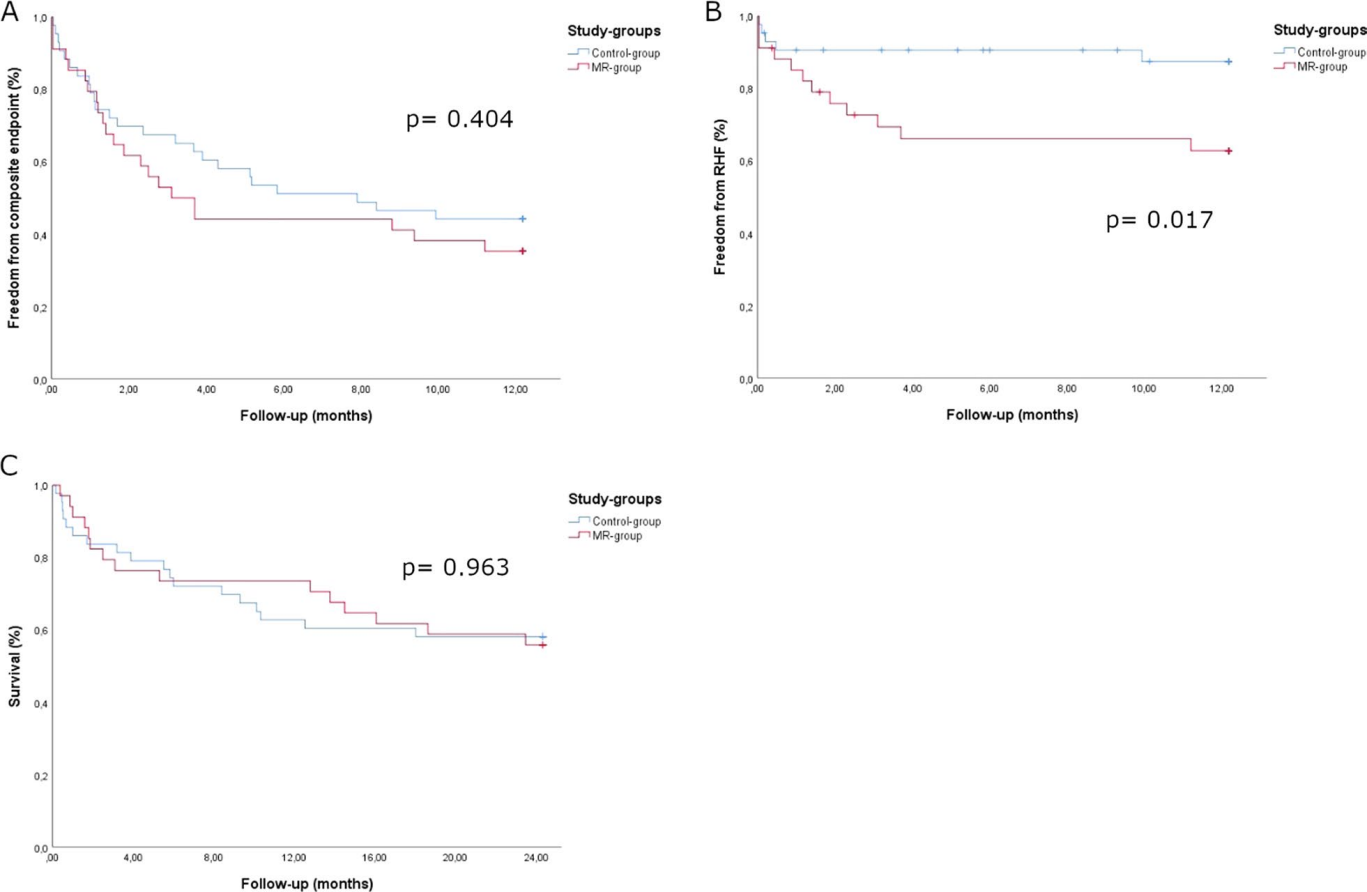
Primary study endpoint, RHF and survival. Kaplan–Meier curves: **A** primary composite outcome of all-cause mortality, stroke, pump-thrombosis, major bleeding and right heart failure (RHF) after 1 year. Secondary outcomes: **B** right-heart failure within first postoperative year and **C** all-cause mortality. *p* values reflecting log-rank test between both study-groups

There was no significant difference regarding the primary composite study endpoint between both study-groups. Freedom from death, stroke, pump-thrombosis, major bleeding or RHF within the first year after LVAD implantation occurred in 12 (35.3%) patients in the ***MR-group*** versus 19 (44.2%) patients in the ***Control-group*** (*p* = 0.404) (Fig. 2A).

### Overall survival and secondary adverse events

Overall survival during follow-up was 57.1% (55.9% ***MR-group*** vs. 58.1% ***Control-group***; *p* = 0.963) (Fig. 2C). The main cause of death was persisting RHF leading to multi-organ failure. Furthermore, RHF within the first year after LVAD implantation occurred more frequently in the ***MR-group*** (35.3% ***MR-group*** vs. 11.6% ***Control-group***; *p* = 0.017) (Fig. 2B). The rate of HTx within the first 2 years after LVAD implantation was comparable between both study-groups (23.5% ***MR-group*** vs. 9.3% ***Control-group***; *p* = 0.123)**.**

### Echocardiographic outcome at 1-year postoperatively

**Fig. 3:**
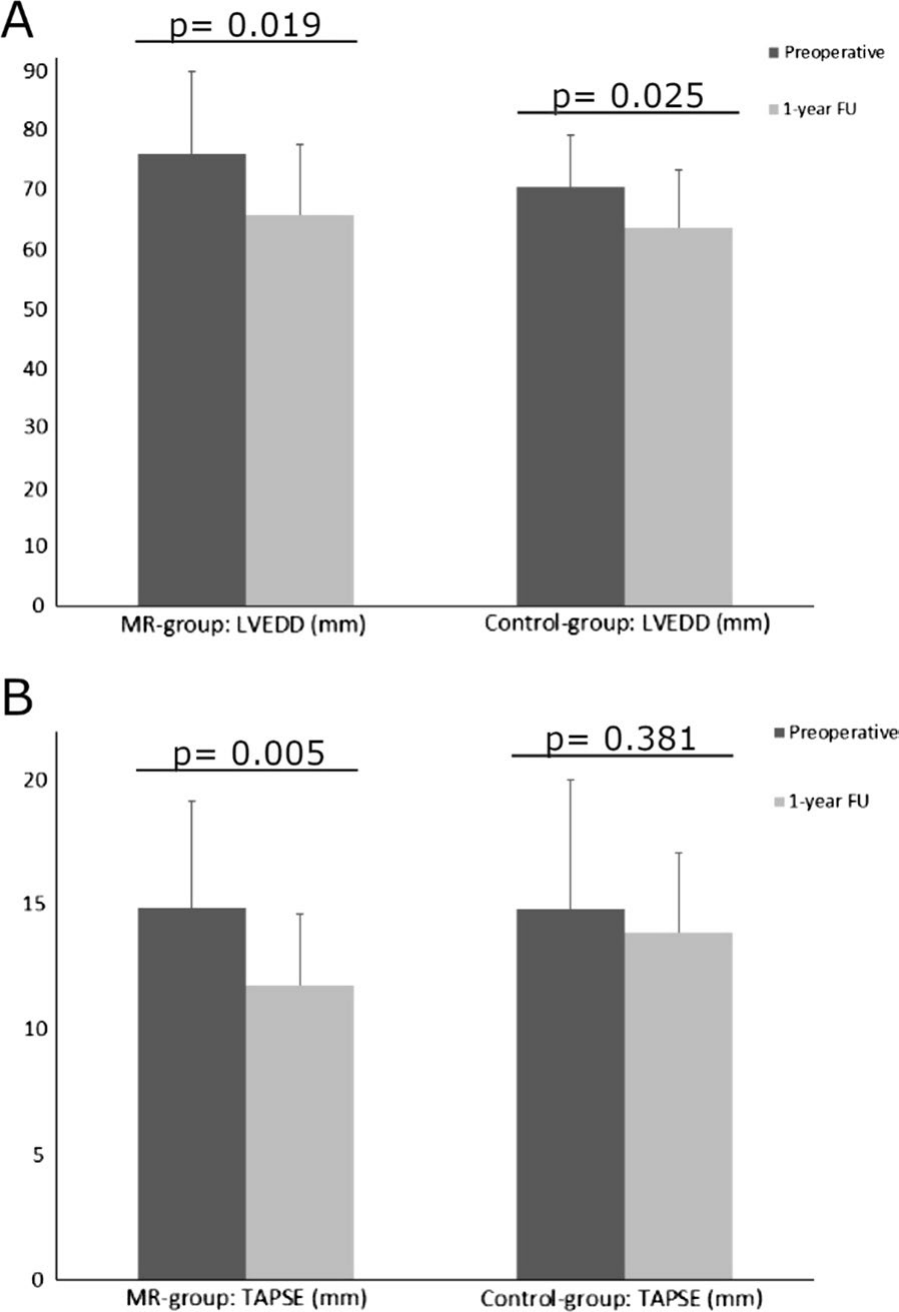
Echocardiographic follow-up. Echocardiographic outcome parameters 1 year after LVAD implantation in comparison to preoperative values. **A** LVEDD: left ventricular end-diastolic diameter. **B** TAPSE: tricuspid annular plane systolic excursion

1-year after LVAD-implantation LVEDD decreased significantly in both study-groups in comparison to preoperative (baseline) values (from 76 ± 14 mm (baseline) to 66 ± 12 mm (1y-FU), *p* = 0.019 ***MR-group***; from 70 ± 9 mm (baseline) to 64 ± 10 mm (1y-FU), *p* = 0.025 ***Control-group***) (Fig. 3A), whereas the extent of LV-dysfunction remained unchanged (Table 4). The prevalence of residual MR ≥ 2 was significantly higher in the ***MR-group*** in comparison to the ***Control-group*** (8 (34.8%) ***MR-group*** vs. 2 (7.4%) ***Control-group***; *p* = 0.016) (Table 4). 1-year postoperatively, RV-function, measured as tricuspid annular plane systolic excursion (TAPSE), was significantly reduced in the ***MR-group*** in comparison to baseline values (TAPSE 14.9 ± 4 mm vs. 11.8 ± 3 mm; *p* = 0.005), whereas no differences occurred in the ***Control-group*** (TAPSE 14.9 ± 5 mm vs. 13.9 ± 3 mm; *p* = 0.381) (Fig. 3B). Furthermore, RV-function, measured as TAPSE, was significantly lower in the ***MR-group*** in comparison to the ***Control-group***, 1 year after LVAD implantation (Table 4).

**Table 4:** 1-year follow-up outcome

Variables	MR-group (n = 23)	Control-group (n = 27)	*p* value
LVEF (%), mean ± SD	20.9 ± 4.8	22.5 ± 5.9	0.278
LVEDD (mm), mean ± SD	65.7 ± 11.8	63.5 ± 9.7	0.568
TAPSE (mm), mean ± SD	11.8 ± 2.9	13.9 ± 3.2	0.029
Residual mitral regurgitation ≥ 3, % (n)	8 (34.8)	2 (7.4)	0.016
Tricuspid regurgitation ≥ 3, n (%)	9 (39.1)	2 (7.4)	0.007
NYHA III–IV, n (%)	17 (73.9)	6 (24.0)	< 0.001
6 MWT (meter), mean ± SD	363 ± 163	405 ± 86	0.316
Serum NT-proBNP level (pg/dl), mean ± SD	7297 ± 10,682	1988 ± 1590	0.024
Serum GOT level (U/l), mean ± SD	27.3 ± 18.7	28.0 ± 19.6	0.898
Serum GPT level (U/l), mean ± SD	30.0 ± 26.2	25.3 ± 13.8	0.442
Serum bilirubin level (mg/dl), mean ± SD	0.9 ± 0.6	0.8 ± 0.4	0.503
Serum creatinin level (mg/dl), mean ± SD	1.8 ± 0.9	1.6 ± 0.4	0.490

*NYHA* New York Heart Association, *6 MWT* 6-min walk test, *NT-pro-BNP* N-terminal pro-B natriuretic peptide, *GOT* glutamic oxaloacetic transaminase, *GPT* glutamic pyruvic transaminase

### Functional outcome at 1-year postoperatively

At 1-year follow-up, a significant improvement in NYHA functional class was found in the whole study-cohort (100% NYHA III/IV preoperatively vs. 46% NYHA III/IV postoperatively (excluded are 25 patients who died and two patients who underwent HTx during the first postoperative year; *p* < 0.001) (Table 4). Notably, significantly more patients within the ***MR-group*** (73.9%) were categorized as NYHA III/IV at 1-year-follow up in comparison to the ***Control-group*** (22.2%) (*p* < 0.001) (Table 4). Accordingly, serum levels of NT-proBNP, which decreased significantly 1-year after LVAD implantation in the ***Control-group*** (9692 ± 8816 pg/ml at baseline vs. 1988 ± 1590 pg/ml at 1-year; *p* < 0.001), remained significantly higher in the ***MR-group*** (10,336 ± 8360 pg/ml at baseline vs. 7297 ± 10,682 pg/ml at 1-year; *p* = 0.262) (*p* = 0.024) (Table 4).

Whereas serum levels of GOT, GPT and bilirubin decreased significantly within the ***Control-group***, the decline of serum levels of GPT and bilirubin did not reach statistical significance in the ***MR-group*** 1 year after LVAD-implantation (Table 4).

## Discussion

Due to mitral annulus dilatation along with severe leaflet tethering, FMR is a common feature of advanced LV remodeling during the progression of chronic heart failure, leading to an impaired outcome [1, 2]. Despite mechanical LV unloading followed by reverse cardiac remodeling and improved mitral leaflet coaptation [14–16], uncorrected FMR persists in up to 30% of LVAD recipients [17, 21, 27]. Although, routine concomitant mitral valve surgery during LVAD implantation is not recommended, the prognostic impact of uncorrected FMR prior to LVAD implantation remains controversial.

### Study population

As a consequence of advanced LV remodeling, 44.2% of the patients in our study-cohort showed moderate to severe FMR (***MR-group***) prior to LVAD implantation. Consequentially increased left atrial pressure, secondary pulmonary congestion and an increased RV afterload, may result in the development of functional TR (FTR) [28]. Of note, the prevalence of FTR was increased within the ***MR-group***. Nevertheless, due to its dependence on the patient’s volume status and a missing standardization, the assessment of FTR and its potential consequences remains challenging, particularly in the context of acute cardiogenic shock and the presence of temporary mechanical circulatory support devices. Apart from an increased prevalence of ischemic cardiomyopathy in the ***Control-group***, baseline characteristics including age, gender and relevant comorbidities were comparable between both study-groups. Notably, preoperative symptoms of congestive heart failure (e.g. NYHA class), as well as signs of secondary end-organ damage (e.g. prevalence of renal failure, previous hemodialysis and hepatic congestion) emerged comparably in both study-groups. Furthermore, the magnitude of systolic LV dysfunction and dilatation was comparable in both study-groups. Despite presumably elevated left-sided filling pressures and pulmonary congestion due to FMR [29] in the ***MR-group***, systolic RV function (measured as TAPSE) prior to LVAD implantation was comparable in both study-groups. Nevertheless, the increased prevalence of significant FTR within the ***MR-group*** might impact the assessment of RV function as severe pendulous volume caused by atrio-ventricular regurgitation potentially leads to hyperdynamic RV function to maintain sufficient stroke volume [30].

### Periprocedural outcome

In accordance with comparable baseline characteristics, periprocedural outcome was similar in both study-groups. Although, tricuspid valve repair was performed more frequently in the ***MR-group***, implantation of a tRVAD due to unsuccessful weaning from CBP, was performed equally in both study-groups. While approximately one third of patients needed short-term MCS prior to LVAD implantation, emphasizing an increased surgical risk of our study patients, there was no procedural mortality within both study-groups.

### Primary study endpoint, survival and secondary adverse events

In accordance with previously published data [16, 18], we found no significant difference regarding the primary composite study endpoint (Fig. 2A). Furthermore, overall survival was similar within both study-groups (Fig. 2C). After a mean follow-up of 24.9 months overall survival was 57.1%, whereas the rate of HTx within the first 2 years after LVAD implantation was 15.6% within the whole study-cohort. Of note, a high proportion of patients, who were included in the current analysis had an increased mortality risk, reflected in the high prevalence of preoperative short-term mechanical circulatory support of 28.6%. Additionally, all of those patients suffered from progressive heart failure or ongoing cardiogenic shock and were classified as INTERMACS class I or II. Furthermore, a high proportion of HVAD implantations within the current analysis might have negatively influenced long-term survival, as there is growing evidence of a higher frequency of neurological adverse events and mortality among HVAD recipients as compared to other commercially available durable LVADs [31].

Although we found a comparable RV function prior to LVAD implantation, RHF within the first postoperative year after LVAD implantation occurred more frequently in the ***MR-group*** (Fig. 2B). Despite mechanical unloading after LVAD implantation, residual FMR might lead to persisting pulmonary congestion and subsequently increased RV afterload [32]. Additionally, the preoperatively increased rate of significant FTR might emphasize an increased susceptibility for RHF in the ***MR-group*** [33], as the evaluation of RV function and the potential risk of RHF after LVAD implantation remains challenging.

### Echocardiographic and functional outcome

One year after LVAD implantation mechanical unloading leads to a significant reduction of LV dilatation, in both study-groups (Fig. 3A). Irrespectively of the remarkable LV size reduction, uncorrected moderate to severe FMR prior to LVAD implantation persisted in 35% of patients in the ***MR-group***. In addition to mitral annulus dilatation, advanced LV remodeling leads to apicolateral displacement of both papillary muscles, resulting in severe mitral leaflet tethering. Interestingly, severe uncorrected leaflet tethering leading to relevant FMR, seems to be associated with persisting FMR after LVAD implantation [17]. Therefore, one could speculate, that patients with persisting FMR after LVAD implantation, exhibited aggravated LV remodeling and consecutive leaflet tethering prior to LVAD implantation, despite a comparable extend of LV dilatation.

In accordance with an increased rate of RHF in the ***MR-group***, RV function was significantly reduced 1 year after LVAD implantation in the ***MR-group***, whereas RV function remained unchanged in the ***Control-group*** (Fig. 3B). Furthermore, TAPSE, was significantly lower in the ***MR-group*** in comparison to the ***Control-group***, 1 year after LVAD implantation (Table 4). Increased RV afterload due to persisting MR after LVAD implantation, presumably promotes the impairment of RV function leading to an increased rate of RHF [21]. Whereas serum markers for hepatic congestion due to RV dysfunction decreased significantly within the ***Control-group***, the decline of serum levels of GPT and bilirubin did not reach statistical significance in the ***MR-group*** 1 year after LVAD-implantation (Table 4).

In the treatment of symptomatic advanced heart failure “destination LVAD therapy” evolved as an alternative to HTx. Consequently, the improvement of heart failure symptoms as well as the reduction of adverse cardiac events acquired an increased importance. Herein, we could demonstrate, that 1 year after LVAD implantation a significant improvement in NYHA functional class was found in the whole study-cohort (100% NYHA III/IV preoperatively vs. 46% NYHA III/IV postoperatively; *p* < 0.001) (Table 4). Notably, significantly more patients within the ***MR-group*** (73.9%) were categorized as NYHA III/IV at 1-year-follow up in comparison to the ***Control-group*** (22.2%) (*p* < 0.001) (Table 4). Accordingly, serum levels of NT-proBNP, which decreased 1-year after LVAD implantation in the ***Control-group*** remained significantly higher in the ***MR-group*** (Table 4). In addition to significantly reduced RV function, FMR prior to LVAD implantation seems to be associated with persisting symptoms of heart failure. Therefore, to further improve long-term patient outcome, concomitant mitral valve surgery in addition to LVAD implantation, especially addressing mitral leaflet tethering [34], might be an option for highly selected heart failure patients with an increased risk of persisting FMR [35] despite LVAD implantation.

### Study limitations

We are aware of a limited patient-cohort treated in a single-center. Furthermore, up to now our follow-up period is limited to 2 years. Nevertheless, due to the inclusion of all consecutive LVAD recipients at our institution and the collection of pre-, peri- and postoperative data using a standardized protocol, a potential selection bias is neglectable. Furthermore, comparable baseline characteristics emphasize the homogeneity of our study-cohort.

## Conclusion

Despite significant LV remodeling due to mechanical unloading after LVAD implantation, residual FMR persisted in one third of the patients. Preoperative uncorrected FMR ≥ 2 prior to LVAD implantation did not affect overall survival, nevertheless it was associated with an impaired RV function and increased rates of RHF within the first postoperative year. Furthermore, preoperative FMR was associated with persistent symptoms of heart failure and increased levels of natriuretic peptide. Therefore, detailed preoperative echocardiographic assessment regarding FMR characteristics (e.g., extend of leaflet tethering) as well as RV function, including the evaluation of possible concomitant mitral valve surgery, are recommendable to further improve patient outcome.

## Data Availability

The datasets used for this study are available from the corresponding authors on reasonable request.

## Abbreviations

AR: Aortic regurgitation
CABG: Coronary artery bypass grafting
CBP: Cardiopulmonary bypass
FMR: Functional mitral regurgitation
GDMT: Guideline-directed medical therapy
GOT: Glutamic oxaloacetic transaminase
GPT: Glutamic pyruvic transaminase
LV: Left ventricle
LVAD: Left ventricular assist device
LVEDD: Left ventricular end-diastolic diameter
LVEF: Left ventricular ejection fraction
MCS: Mechanical circulatory support
MR: Mitral regurgitation
NT-pro-BNP: N-terminal pro-B natriuretic peptide
NYHA: New York Heart Association
RHF: Right heart failure
RV: Right ventricle
TAPSE: Tricuspid annular plane systolic excursion
TR: Tricuspid regurgitation
tRVAD: Temporary right ventricular assist device
6 MWT: 6-Min walk test
